# Predicting risk of early discontinuation of exclusive breastfeeding at a Brazilian referral hospital for high-risk neonates and infants: a decision-tree analysis

**DOI:** 10.1186/s13006-020-00349-x

**Published:** 2021-01-04

**Authors:** Maíra Domingues Bernardes Silva, Raquel de Vasconcellos Carvalhaes de Oliveira, Davi da Silveira Barroso Alves, Enirtes Caetano Prates Melo

**Affiliations:** 1 Human Milk Bank at the National Institute of Women, Children and Adolescents Health Fernandes Figueira (IFF) of the Oswaldo Cruz Foundation (FIOCRUZ), Rio de Janeiro, RJ Brazil; 2 National Institute of Infectious Diseases (FIOCRUZ), Rio de Janeiro, RJ Brazil; 3 Federal University of the State of Rio de Janeiro (UNIRIO), Rio de Janeiro, RJ Brazil; 4 National School of Public Health (FIOCRUZ), Rio de Janeiro, RJ Brazil

## Abstract

**Background:**

Determinants at several levels may affect breastfeeding practices. Besides the known historical, socio-economic, cultural, and individual factors, other components also pose major challenges to breastfeeding. Predicting existing patterns and identifying modifiable components are important for achieving optimal results as early as possible, especially in the most vulnerable population. The goal of this study was building a tree-based analysis to determine the variables that can predict the pattern of breastfeeding at hospital discharge and at 3 and 6 months of age in a referral center for high-risk infants.

**Methods:**

This prospective, longitudinal study included 1003 infants and was conducted at a high-risk public hospital in the following three phases: hospital admission, first visit after discharge, and monthly telephone interview until the sixth month of the infant’s life. Independent variables were sorted into four groups: factors related to the newborn infant, mother, health service, and breastfeeding. The outcome was breastfeeding as per *the categories established by the World Health Organization (*WHO). For this study, we performed an exploratory analysis at hospital discharge and at 3 and at 6 months of age in two stages, as follows: (i) determining the frequencies of baseline characteristics stratified by breastfeeding indicators in the three mentioned periods and (ii) decision-tree analysis.

**Results:**

The prevalence of exclusive breastfeeding (EBF) was 65.2% at hospital discharge, 51% at 3 months, and 20.6% at 6 months. At hospital discharge and the sixth month, the length of hospital stay was the most important predictor of feeding practices, also relevant at the third month. Besides the mother’s and child’s characteristics (multiple births, maternal age, and parity), the social context, work, feeding practice during hospitalization, and hospital practices and policies on breastfeeding influenced the breastfeeding rates.

**Conclusions:**

The combination algorithm of decision trees (a machine learning technique) provides a better understanding of the risk predictors of breastfeeding cessation in a setting with a large variability in expositions. Decision trees may provide a basis for recommendations aimed at this high-risk population, within the Brazilian context, in light of the hospital stay at a neonatal unit and period of continuous feeding practice.

**Supplementary Information:**

The online version contains supplementary material available at 10.1186/s13006-020-00349-x.

## Background

Globally, determinants at several levels may affect breastfeeding practices [1]. In environments subject to clinical vulnerability, besides the several known historical, socio-economic, cultural, and individual factors, other components also pose major challenges to breastfeeding [2, 3]. Brazilian studies, selected in a systematic review [4] on breastfeeding determinants, have not investigated factors associated with breastfeeding in high-risk infants. In addition, such studies were based on regression models (Poisson, logistic, Cox) for statistical analysis [4], a technique also broadly used in the international literature on this field [5].

Traditional regression models are often limited in the exploration of the mutual importance of exposures. Thus, machine learning techniques may be able to investigate the network between exposures and eventually develop decision rules for estimating the risk of early discontinuation of exclusive breastfeeding (EBF) in clinical work. Predicting existing patterns and identifying modifiable components, along with existing studies, are important for reaching the best results as early as possible, especially when dealing with vulnerable populations. Studies using methodologies for predicting situations that might lead to early discontinuation of breastfeeding may help design effective decision-making strategies, especially for subgroups facing major challenges in daily clinical practice.

In the present study, a decision tree model was constructed and validated to determine the variables that can predict the pattern of breastfeeding at hospital discharge and at 3 and 6 months of age, in a referral center for high-risk infants.

## Methods

### Design, setting, and study participants

This was a prospective cohort study conducted in Rio de Janeiro, Brazil, at the National Institute of Women, Children and Adolescents’ Health Fernandes Figueira (IFF) of the Oswaldo Cruz Foundation (FIOCRUZ), a public referral hospital for fetuses, neonates, and infants at high risk. This public hospital attends to about 1000 deliveries per year, is accredited as the Baby-Friendly Hospital Initiative (BFHI), and receives newborns and children with congenital malformations or genetic syndromes from all over Brazil.

**Fig. 1:**
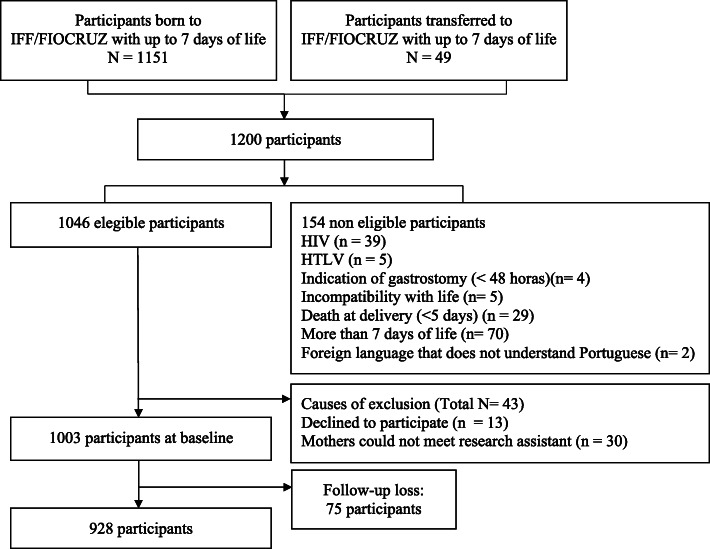
Flowchart of participant selection. Note: FIOCRUZ = Oswaldo Cruz Foundation; HIV = Human Immunodeficiency Virus; HTLV = Human T-cell Lymphotropic Virus; IFF = National Institute of Women, Children and Adolescents Health Fernandes Figueira

The study population included all neonates delivered or transferred to the referral center from March 2017 to April 2018. Of the 1200 eligible participants, 154 were excluded due to non-eligibility, 30 could not meet the research assistant, and the other 13 nursing mothers declined to participate in the study. Figure 1 illustrates the flowchart of the selection process of the participants in this study. Details about participants, setting, and procedures have been published elsewhere [6].

### Data collection

In all, 1003 infants were enrolled in the longitudinal study of breastfeeding conducted in a Brazilian referral center for high-risk fetuses, neonates, and infants. Each infant was followed up for up to 6 months of life. The end of the follow-up period was October 2018.

This study was developed in three phases: (a) in the first phase, data were obtained from interviews with mothers and medical records; (b) in the second phase, the mothers were interviewed during the first visit after hospital discharge; and (c) in the third phase, telephone interviews were conducted every month until the sixth month of the infant’s life. Regarding this last phase, up to 10 telephone contact attempts were made with each participant each month to minimize loss to follow-up. Data were collected through a web application developed for the research, which could be accessed by using a mobile and/or computer with internet access. A control and quality assurance process was established for data collection, as described elsewhere [6].

### Data measures

The outcome was investigated every month during telephone interviews and was assessed by the question “During the month preceding the interview, what foods have you offered to your children?” The response categories were mother’s milk, another type of milk, water, tea, juice, fruits, and any other foods. The participants were categorized into four groups for the analysis of the outcome, according to the set of indicators used for assessing breastfeeding practices that reflect the guidelines on breastfeeding: exclusive breastfeeding (EBF), i.e., breastfeeding not supplemented with any other fluids or solid foods; predominant breastfeeding, i.e., breastfeeding supplemented with fluids such as water, tea, or fruit juices but not solid or semi-solid foods; partial breastfeeding (PBF), i.e., breastfeeding supplemented with other types of milk, such as infant formula, and solid or semi-solid foods; and non-breastfed (NBF), i.e., no breastfeeding [7]. Owing to the low prevalence of “predominant breastfeeding” in the third and sixth months, it was not possible to use this category alone in the analysis. Therefore, the categories “exclusive breastfeeding” and “predominant breastfeeding” were combined and renamed as “exclusive or predominant breastfeeding” (EPB).

The covariates used in the analysis represented (a) maternal factors – “maternal education,” “tobacco use during pregnancy,” “parity and previous experience of breastfeeding,” “presence of partner at home,” “household income” (as compared to the reference value of the prevailing monthly minimum wage in Brazil, which is the minimum payment value per month for formal employees, as prescribed by law), “gestational morbidity,” “maternal work and maternity leave,” “maternal age,” and “breastfeeding difficulties”; (b) child-related factors – “multiples at births,” “birthweight,” “gestational age,” “perinatal morbidity,” and “surgical morbidity at birth”; and (c) health service-related factors – “length of hospital stay,” “use of pasteurized donor human milk,” “infant received formula,” “use of cup-feeding,” “skin-to-skin contact in the delivery room,” “place of hospital admission” (maternity ward or neonatal intensive care unit), “breastfeeding advising during prenatal period,” “use of a pacifier,” and “mode of delivery.” In the third and sixth months, the variables “hospital readmission,” “feeding practice at hospital discharge,” and “breastfeeding difficulties in the month prior to the monthly interview” were added.

### Data analysis

The first stage involved a bivariate analysis of maternal and neonatal characteristics according to the feeding practices at hospital discharge and at 3 and 6 months of age. The associations were checked by Pearson’s chi-squared tests. When the expected frequency was lower than five in the contingency tables, Fisher’s exact test was applied. The Dunn test was applied for the analysis of variables “length of hospital stay” and “feeding practice” at 3 and 6 months of age. Since the use of *p* - values is not recommended in large samples [8], confidence intervals (CI) were provided as a measurement of uncertainty, and *p* - values were considered as additional information. Besides, differences of at least 10 percentage points (pp.) among feeding practices were considered among the included and excluded participants, suggesting a difference.

In the second stage, decision-tree models were adjusted by using the *CART* algorithm [9] at hospital discharge and in the third and sixth months, with the indicators for assessing breastfeeding practices used as dependent variables. The decision-tree models are machine learning algorithms that define the rules for recursive binary divisions (binary because the node parents are always divided exactly into two child nodes and recursive because the process can be repeated by treating each child node as a parent node), expressed in values or categories of independent variables, with the purpose of defining the prediction of a categorical variable, represented in decision-tree graphs [9].

From the total set of analyzed data, i.e., the “root” of the tree, the algorithm selects predictor variables for each possible partition, the “nodes,” using an impurity measure defined according to the category distribution of the predicted variables in subgroups derived from the possible divisions, generating a “branch” until a minimum number of elements in the subdivision is reached or until there are no gains in prediction [10, 11]. The tree “leaves” represent categories of the recurrent outcome resulting from these divisions.

There are two important reasons to consider variable selection using decision trees when developing risk predictions. First, limiting the number of inputs to be supplied by the user may increase the utilization of a prediction tool. Second, the elimination of variables that are not predictive may improve prediction accuracy [12].

A 10-fold cross-validation process with three repetitions was used for the adjustment of the hyperparameter of the maximum depth for each of the three models from which the most accurate resultant value was selected. The adjusted models were presented in the form of a decision tree for each period and with at least two informative variables.

The tree is designed with graphic boxes and lines. The predictor of major importance is at the top, and the branches are built according to a decreasing hierarchy of importance until it reaches the leaf. Inside each leaf, located in the lower part of the tree, the most frequent feeding practice is highlighted. The second line presents the probability for each outcome category, in the following sequence: EBF (hospital discharge), EPB (third and sixth months), PBF, and NBF. The last leaf line shows the frequency of participants from that branch.

The participants who were lost to follow-up were excluded from the analysis. From the original sample, 75 children (7.5%) considered for the analysis in the first stage of the study (baseline) did not continue after hospital discharge, so they were excluded from the total number of participants.

The R Foundation for Statistical Computing, version 3.5.2, was used to analyze *the data.* The *rpart* library [11] was used to fit the decision-tree model; the *caret* library [13] was used to tune the max depth parameter with 10-fold cross-validation, and the *rattle* library [14] was used to obtain the decision-tree graphs. This study was approved by the Ethics Committees at IFF/FIOCRUZ, Brazil (Protocol Number: 1.930.996–2017).

## Results

The prevalence of exclusive breastfeeding at discharge was 65.2% (95% CI 62.2,68.3), and 51% at 3 months (95% CI 47.1,54.8); 20.6% (95% CI 16.5,25.0) of the participants were still exclusively breastfed at 6 months postpartum. A few mothers maintained predominant breastfeeding for 3 months (7.1%; 95% CI 3.2,11.0) and 6 months (9.3%; 95% CI 5.2,13.7); therefore, the EPB category had a higher proportion of infants from the “exclusive breastfeeding” category than from the “predominant breastfeeding” category.

Table 1 shows the wide variability in mother and infant characteristics according to the feeding practice at discharge and at 3 and 6 months. The mothers had a mean age of 27 years, ranging from 13 to 46 years; nearly all mothers had planned to breastfeed, and it is important to highlight that over 50% of mothers had some difficulty with breastfeeding before discharge.

**Table 1:** Characteristics of the participants stratified by feeding practice and period. Rio de Janeiro, Brazil, 2018

Characteristics		Hospital discharge			3 months of age			6 months of age		
		% (95% CI)			% (95% CI)			% (95% CI)		
		EBF (***N*** = 495)	PBF (*N* = 208)	NBF (*N* = 54)	EPB (***N*** = 302)	PBF (***N*** = 166)	NBF (***N*** = 58)	EPB (***N*** = 137)	PBF (***N*** = 231)	NBF (***N*** = 91)
Child-related factors										
Sex	female	48.1 (43.6–52.6)	46.6 (39.7–53.7)	48.1 (34.3–62.2)	49.3 (43.6–55.1)	53.0 (45.1–60.8)	39.7 (27.0–53.4)	43.1 (34.6–51.8)	50.6 (44.0–57.3)	42.9 (32.5–53.7)
	male	51.9 (47.4–56.4)	53.4 (46.3–60.3)	51.9 (37.8–65.7)	50.7 (44.9–56.4)	47.0 (39.2–54.9)	60.3 (46.6–73.0)	56.9 (48.2–65.4)	49.4 (42.7–56.0)	57.1 (46.3–67.5)
Multiple births	no	**87.7 (84.5–90.4)**	76.0 (69.6–81.6)	79.6 (66.5–89.4)	**93.0 (89.6–95.6)**	**70.5 (62.9–77.3)**	**74.1 (61.0–84.7)**	**95.6 (90.7–98.4)**	**92.6 (88.5–95.7)**	**71.4 (61.0–80.4)**
	yes	**12.3 (9.6–15.5)**	24.0 (18.4–30.4)	20.4 (10.6–33.5)	**7.0 (4.4–10.4)**	**29.5 (22.7–37.1)**	**25.9 (15.3–39.0)**	**4.4 (1.6–9.3)**	**7.4 (4.3–11.5)**	**28.6 (19.6–39.0)**
Gestational age (weeks)	> 37	**85.7 (82.3–88.6)**	68.8 (62.0–75.0)	42.6 (29.2–56.8)	**89.1 (85.0–92.4)**	**72.3 (64.8–78.9)**	**60.3 (46.6–73.0)**	**89.1 (82.6–93.7)**	**88.3 (83.5–92.2)**	**60.4 (49.6–70.5)**
	< 37	**14.3 (11.4–17.7)**	31.2 (25.0–38.0)	57.4 (43.2–70.8)	**10.9 (7.6–15.0)**	**27.7 (21.1–35.2)**	**39.7 (27.0–53.4)**	**10.9 (6.3–17.4)**	**11.7 (7.8–16.5)**	**39.6 (29.5–50.4)**
Birth weight	ELBW	**0.4 (0.0–1.5)**	0.5 (0.0–2.6)	9.3 (3.1–20.3)	**0.0 (0.0–1.2)**	**1.2 (0.1–4.3)**	**0.0 (0.0–6.2)**	**0.0 (0.0–2.7)**	**0.4 (0.0–2.4)**	**2.2 (0.3–7.7)**
	VLBW	**0.2 (0.0–1.1)**	1.9 (0.5–4.9)	16.7 (7.9–29.3)	**0.7 (0.1–2.4)**	**1.8 (0.4–5.2)**	**5.2 (1.1–14.4)**	**0.7 (0.0–4.0)**	**0.9 (0.1–3.1)**	**5.5 (1.8–12.4)**
	LBW	**10.1 (7.6–13.1)**	27.9 (21.9–34.5)	20.4 (10.6–33.5)	**10.6 (7.4–14.6)**	**21.7 (15.7–28.7)**	**20.7 (11.2–33.4)**	**9.5 (5.1–15.7)**	**10.8 (7.1–15.6)**	**25.3 (16.7–35.5)**
	> 2500 g	**89.3 (86.2–91.9)**	69.7 (63.0–75.9)	53.7 (39.6–67.4)	**88.7 (84.6–92.1)**	**75.3 (68.0–81.7)**	**74.1 (61.0–84.7)**	**89.8 (83.4–94.3)**	**87.9 (83.0–91.8)**	**67.0 (56.4–76.5)**
Perinatal morbidity	no	**69.3 (65.0–73.3)**	48.1 (41.1–55.1)	0.0 (0.0–6.6)	**71.9 (66.4–76.9)**	**54.2 (46.3–62.0)**	**37.9 (25.5–51.6)**	**70.1 (61.7–77.6)**	**70.6 (64.2–76.4)**	**34.1 (24.5–44.7)**
	yes	**30.7 (26.7–35.0)**	51.9 (44.9–58.9)	100.0 (93.4–100.0)	**28.1 (23.1–33.6)**	**45.8 (38.0–53.7)**	**62.1 (48.4–74.5)**	**29.9 (22.4–38.3)**	**29.4 (23.6–35.8)**	**65.9 (55.3–75.5)**
Hospital readmission	no	–	–	–	**95.7 (92.8–97.7)**	**92.8 (87.7–96.2)**	**82.8 (70.6–91.4)**	97.1 (92.7–99.2)	93.9 (90.0–96.6)	91.2 (83.4–96.1)
	yes	–	–	–	**4.3 (2.3–7.2)**	**7.2 (3.8–12.3)**	**17.2 (8.6–29.4)**	2.9 (0.8–7.3)	6.1 (3.4–10.0)	8.8 (3.9–16.6)
Mother-related factors										
Maternal age	< 20 y.o.	10.9 (8.3–14.0)	6.2 (3.4–10.5)	20.4 (10.6–33.5)	10.3 (7.1–14.3)	6.6 (3.4–11.5)	8.6 (2.9–19.0)	8.0 (4.1–13.9)	10.0 (6.4–14.6)	9.9 (4.6–17.9)
	from 20 to 34 y.o.	69.7 (65.4–73.7)	76.0 (69.6–81.6)	59.3 (45.0–72.4)	71.5 (66.1–76.5)	68.7 (61.0–75.6)	74.1 (61.0–84.7)	75.9 (67.9–82.8)	63.6 (57.1–69.8)	73.6 (63.3–82.3)
	> 35 y.o.	19.4 (16.0–23.2)	17.8 (12.8–23.7)	20.4 (10.6–33.5)	18.2 (14.0–23.0)	24.7 (18.3–32.0)	17.2 (8.6–29.4)	16.1 (10.3–23.3)	26.4 (20.8–32.6)	16.5 (9.5–25.7)
Maternal education	up to elementary school	32.3 (28.2–36.6)	34.1 (27.7–41.0)	38.9 (25.9–53.1)	27.8 (22.8–33.2)	33.1 (26.0–40.8)	31.0 (19.5–44.5)	27.0 (19.8–35.3)	25.5 (20.0–31.7)	31.9 (22.5–42.5)
	secondary school or higher	67.7 (63.4–71.8)	65.9 (59.0–72.3)	61.1 (46.9–74.1)	72.2 (66.8–77.2)	66.9 (59.2–74.0)	69.0 (55.5–80.5)	73.0 (64.7–80.2)	74.5 (68.3–80.0)	68.1 (57.5–77.5)
Household income ^a^	less than $576	37.8 (33.5–42.2)	38.9 (32.3–45.9)	51.9 (37.8–65.7)	37.1 (31.6–42.8)	36.1 (28.8–44.0)	46.6 (33.3–60.1)	41.6 (33.3–50.3)	34.2 (28.1–40.7)	38.5 (28.4–49.2)
	over $576	62.2 (57.8–66.5)	61.1 (54.1–67.7)	48.1 (34.3–62.2)	62.9 (57.2–68.4)	63.9 (56.0–71.2)	53.4 (39.9–66.7)	58.4 (49.7–66.7)	65.8 (59.3–71.9)	61.5 (50.8–71.6)
Parity and PEBF	primiparous	49.5 (45.0–54.0)	45.2 (38.3–52.2)	63.0 (48.7–75.7)	51.3 (45.5–57.1)	47.6 (39.8–55.5)	43.1 (30.2–56.8)	54.0 (45.3–62.6)	50.2 (43.6–56.8)	45.1 (34.6–55.8)
	multiparous with PEBF	46.1 (41.6–50.6)	47.1 (40.2–54.1)	35.2 (22.7–49.4)	45.0 (39.3–50.8)	46.4 (38.6–54.3)	41.4 (28.6–55.1)	43.1 (34.6–51.8)	44.6 (38.1–51.2)	42.9 (32.5–53.7)
	multiparous without PEBF	4.4 (2.8–6.7)	7.7 (4.5–12.2)	1.9 (0.0–9.9)	3.6 (1.8–6.4)	6.0 (2.9–10.8)	15.5 (7.3–27.4)	2.9 (0.8–7.3)	5.2 (2.7–8.9)	12.1 (6.2–20.6)
Gestational morbidity	no	53.5 (49.0–58.0)	46.2 (39.2–53.2)	48.1 (34.3–62.2)	54.0 (48.2–59.7)	54.2 (46.3–62.0)	53.4 (39.9–66.7)	57.7 (48.9–66.1)	49.8 (43.2–56.4)	57.1 (46.3–67.5)
	yes	46.5 (42.0–51.0)	53.8 (46.8–60.8)	51.9 (37.8–65.7)	46.0 (40.3–51.8)	45.8 (38.0–53.7)	46.6 (33.3–60.1)	42.3 (33.9–51.1)	50.2 (43.6–56.8)	42.9 (32.5–53.7)
Maternal work and maternity leave	does not work	48.3 (43.8–52.8)	51.4 (44.4–58.4)	57.4 (43.2–70.8)	47.7 (41.9–53.5)	48.2 (40.4–56.1)	65.5 (51.9–77.5)	54.0 (45.3–62.6)	42.4 (36.0–49.1)	59.3 (48.5–69.5)
	works from home	4.8 (3.1–7.1)	5.3 (2.7–9.3)	3.7 (0.5–12.7)	4.0 (2.1–6.8)	9.0 (5.1–14.5)	0.0 (0.0–6.2)	4.4 (1.6–9.3)	6.1 (3.4–10.0)	3.3 (0.7–9.3)
	ML up to 6 mos	6.1 (4.1–8.5)	3.8 (1.7–7.4)	3.7 (0.5–12.7)	7.3 (4.6–10.8)	4.2 (1.7–8.5)	5.2 (1.1–14.4)	8.0 (4.1–13.9)	6.1 (3.4–10.0)	2.2 (0.3–7.7)
	ML up to 4 mos	29.1 (25.1–33.3)	27.4 (21.5–34.0)	16.7 (7.9–29.3)	27.8 (22.8–33.2)	25.9 (19.4–33.3)	24.1 (13.9–37.2)	19.0 (12.8–26.6)	33.3 (27.3–39.8)	23.1 (14.9–33.1)
	works without ML benefits	11.7 (9.0–14.9)	12.0 (7.9–17.2)	18.5 (9.3–31.4)	13.2 (9.6–17.6)	12.7 (8.0–18.7)	5.2 (1.1–14.4)	14.6 (9.2–21.6)	12.1 (8.2–17.0)	12.1 (6.2–20.6)
Health service-related factors										
Mode of delivery	cesarean	**52.7 (48.2–57.2)**	74.0 (67.5–79.9)	81.5 (68.6–90.7)	**53.0 (47.2–58.7)**	**66.3 (58.5–73.4)**	**74.1 (61.0–84.7)**	51.1 (42.4–59.7)	55.4 (48.8–61.9)	70.3 (59.8–79.5)
	transpelvic	**47.3 (42.8–51.8)**	26.0 (20.1–32.5)	18.5 (9.3–31.4)	**47.0 (41.3–52.8)**	**33.7 (26.6–41.5)**	**25.9 (15.3–39.0)**	48.9 (40.3–57.6)	44.6 (38.1–51.2)	29.7 (20.5–40.2)
Use of a pacifier	no	**93.7 (91.2–95.7)**	77.9 (71.6–83.3)	35.2 (22.7–49.4)	**92.7 (89.2–95.4)**	**84.3 (77.9–89.5)**	**69.0 (55.5–80.5)**	**94.9 (89.8–97.9)**	**89.2 (84.4–92.9)**	**67.0 (56.4–76.5)**
	yes	**6.3 (4.3–8.8)**	22.1 (16.7–28.4)	64.8 (50.6–77.3)	**7.3 (4.6–10.8)**	**15.7 (10.5–22.1)**	**31.0 (19.5–44.5)**	**5.1 (2.1–10.2)**	**10.8 (7.1–15.6)**	**33.0 (23.5–43.6)**
Skin-to-skin contact in the delivery room	yes	**55.6 (51.1–60.0)**	34.6 (28.2–41.5)	5.6 (1.2–15.4)	**54.0 (48.2–59.7)**	**44.6 (36.9–52.5)**	**32.8 (21.0–46.3)**	**56.2 (47.5–64.7)**	**51.5 (44.9–58.1)**	**34.1 (24.5–44.7)**
	no	**44.4 (40.0–48.9)**	65.4 (58.5–71.8)	94.4 (84.6–98.8)	**46.0 (40.3–51.8)**	**55.4 (47.5–63.1)**	**67.2 (53.7–79.0)**	**43.8 (35.3–52.5)**	**48.5 (41.9–55.1)**	**65.9 (55.3–75.5)**
BF difficulties	no	**44.6 (40.2–49.1)**	38.5 (31.8–45.4)	7.4 (2.1–17.9)	**46.0 (40.3–51.8)**	**39.2 (31.7–47.0)**	**20.7 (11.2–33.4)**	48.9 (40.3–57.6)	44.6 (38.1–51.2)	28.6 (19.6–39.0)
	yes	**55.4 (50.9–59.8)**	61.5 (54.6–68.2)	92.6 (82.1–97.9)	**54.0 (48.2–59.7)**	**60.8 (53.0–68.3)**	**79.3 (66.6–88.8)**	51.1 (42.4–59.7)	55.4 (48.8–61.9)	71.4 (61.0–80.4)
Use of pasteurized donor human milk	no	**44.0 (39.6–48.5)**	50.0 (43.0–57.0)	1.9 (0.0–9.9)	**51.7 (45.9–57.4)**	**31.9 (24.9–39.6)**	**24.1 (13.9–37.2)**	**56.2 (47.5–64.7)**	**47.2 (40.6–53.8)**	**25.3 (16.7–35.5)**
	yes	**56.0 (51.5–60.4)**	50.0 (43.0–57.0)	98.1 (90.1–100.0)	**48.3 (42.6–54.1)**	**68.1 (60.4–75.1)**	**75.9 (62.8–86.1)**	**43.8 (35.3–52.5)**	**52.8 (46.2–59.4)**	**74.7 (64.5–83.3)**
Use of cup-feeding	no	**44.2 (39.8–48.7)**	13.0 (8.7–18.3)	53.7 (39.6–67.4)	36.4 (31.0–42.1)	27.1 (20.5–34.5)	27.6 (16.7–40.9)	39.4 (31.2–48.1)	35.1 (28.9–41.6)	38.5 (28.4–49.2)
	yes	**55.8 (51.3–60.2)**	87.0 (81.7–91.3)	46.3 (32.6–60.4)	63.6 (57.9–69.0)	72.9 (65.5–79.5)	72.4 (59.1–83.3)	60.6 (51.9–68.8)	64.9 (58.4–71.1)	61.5 (50.8–71.6)
NB received infant formula	no	**96.8 (94.8–98.1)**	3.8 (1.7–7.4)	0.0 (0.0–6.6)	**76.2 (70.9–80.9)**	**60.8 (53.0–68.3)**	**43.1 (30.2–56.8)**	**73.7 (65.5–80.9)**	**71.4 (65.1–77.2)**	**42.9 (32.5–53.7)**
	yes	**3.2 (1.9–5.2)**	96.2 (92.6–98.3)	100.0 (93.4–100.0)	**23.8 (19.1–29.1)**	**39.2 (31.7–47.0)**	**56.9 (43.2–69.8)**	**26.3 (19.1–34.5)**	**28.6 (22.8–34.9)**	**57.1 (46.3–67.5)**
Place admission	maternity ward	**82.2 (78.6–85.5)**	57.2 (50.2–64.0)	0.0 (0.0–6.6)	**81.5 (76.6–85.7)**	**66.3 (58.5–73.4)**	**48.3 (35.0–61.8)**	**81.0 (73.4–87.2)**	**77.9 (72.0–83.1)**	**46.2 (35.6–56.9)**
	NICU	**17.8 (14.5–21.4)**	42.8 (36.0–49.8)	100.0 (93.4–100.0)	**18.5 (14.3–23.4)**	**33.7 (26.6–41.5)**	**51.7 (38.2–65.0)**	**19.0 (12.8–26.6)**	**22.1 (16.9–28.0)**	**53.8 (43.1–64.4)**
Feeding practice at hospital discharge	EBF	–	–	–	**75.8 (70.6–80.5)**	**62.7 (54.8–70.0)**	**43.1 (30.2–56.8)**	**72.3 (64.0–79.6)**	**73.2 (67.0–78.8)**	**44.0 (33.6–54.8)**
	PBF	–	–	–	**23.2 (18.5–28.4)**	**32.5 (25.5–40.2)**	**36.2 (24.0–49.9)**	**26.3 (19.1–34.5)**	**24.2 (18.9–30.3)**	**35.2 (25.4–45.9)**
	NBF	–	–	–	**1.0 (0.2–2.9)**	**4.8 (2.1–9.3)**	**20.7 (11.2–33.4)**	**1.5 (0.2–5.2)**	**2.6 (1.0–5.6)**	**20.9 (13.1–30.7)**

Note: ^a^ Household income (expressed in comparison to a reference value of two Brazilian monthly minimum wages at the time of the perinatal interview). ‘Minimum wage’ refers to the monthly minimum wage, as established by law, for formal employees in Brazil. [http://www.planalto.gov.br/ccivil_03/_Ato2015-2018/2016/Decreto/D8948.htm]; [http://receita.economia.gov.br/orientacao/tributaria/declaracoes-e-demonstrativos/ecf-escrituracao-contabil-fiscal/taxas-de-cambio-incluindo-valor-do-dolar-para-fins-fiscais-irpj-AC-anteriores]

Bold percentages refer to statistical significance (*p*-value < 0.005) and were based on Chi-square and Fisher tests

*EBF* Exclusive breastfeeding. *PBF* Partial breastfeeding. *EPB* Exclusive or predominant breastfeeding. *NBF* Non-breastfed. *CI* Confidence interval. *ELBW* Extremely low birth weight. *VLBW* Very low birth weight. *LBW* Low birth weight. *Y.O.* years old. *PEBF* Previous experience of breastfeeding. *ML* Maternity leave. *MOS* Months. *BF* Breastfeeding. *NB* Newborn. *NICU* Neonatal Intensive Care Unit

Of the infants, 17 (1.7%) had extremely low birthweight, 21 (2.1%) had very low birthweight, 159 (15.9%) had low birthweight, 226 (22.5%) were preterm, and 149 (14.9%) were multiples (twins, triplets, and quadruplets).

Further, 32% of the infants were admitted to the neonatal or neosurgical intensive care unit (NICU), for a mean of 11 days (ranging from 2 to 150 days); 417 (42%) had perinatal morbidity, of which 189 (18.8%) had surgical anomalies and 11 (1.1%) had genetic syndromes such as Down, Werdnig-Hoffmann, Turner, and Beckwith Wiedmann syndromes.

After reassessing all the sample for data checking and disregarding cases with missing data in the three periods of the study, the analysis included data on 757 participants at hospital discharge, 526 participants in the third month, and 459 participants in the sixth month. When assessing the groups of participants who were included in the study and those who were excluded due to missing data, there were differences in the social determinants of “maternal age,” “maternal work and maternity leave,” and “maternal education” between these groups (Additional file 1).

**Fig. 2:**
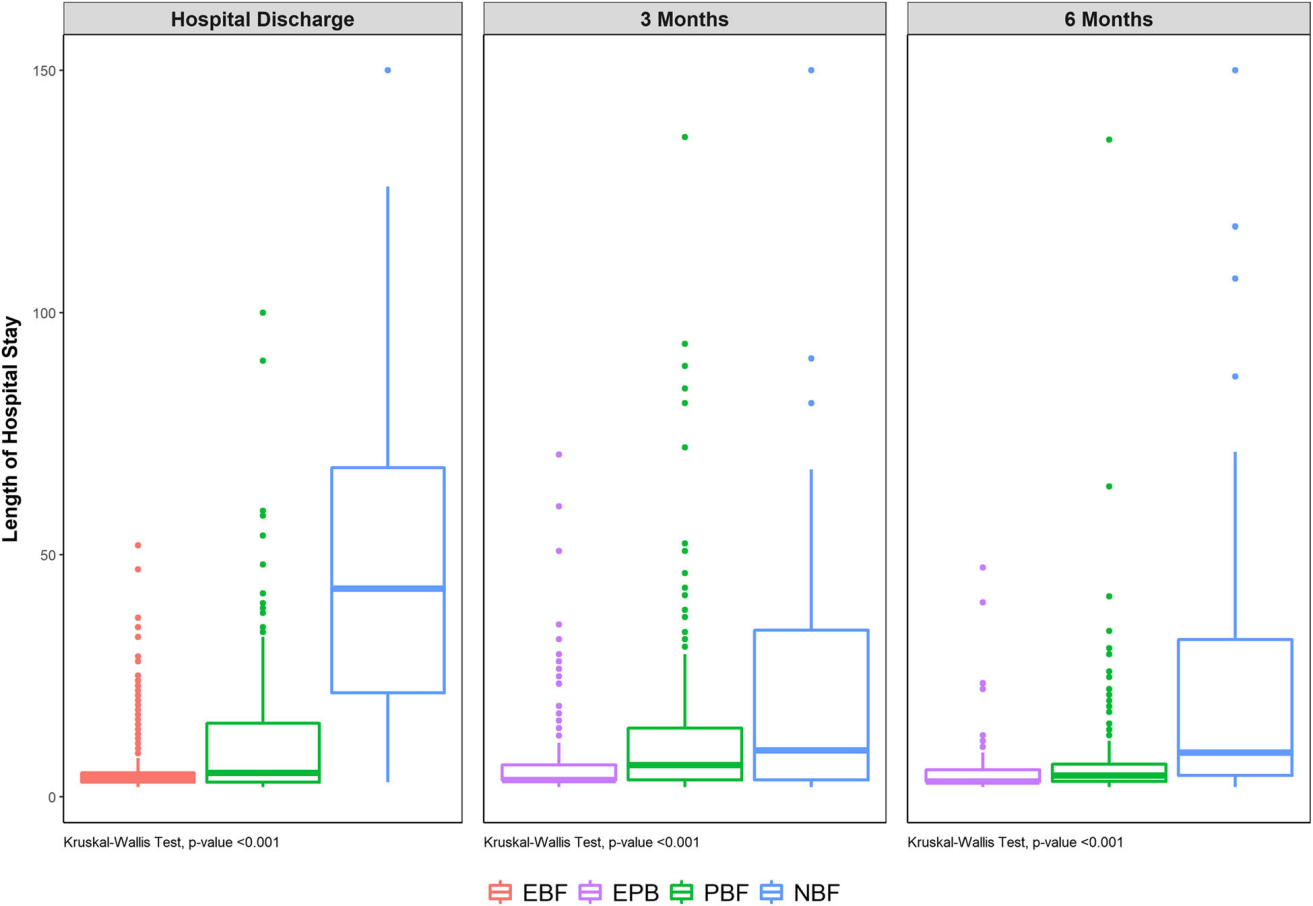
Boxplot of median length of hospital stay regarding feeding practice at hospital discharge, in the third and in the sixth month of life. Note: EBF = Exclusive Breastfeeding; EPB = Exclusive or Predominant Breastfeeding. PBF = Partial Breastfeeding. NBF = Non-Breastfed. The length of hospital stay was measured in days

The median “length of hospital stay” gradually increased from EBF to NBF during the three analyzed periods. The median increment in the NBF group (43 days) was 10-fold greater than that observed in the EBF group (4 days) at discharge and approximately two-to-three times greater in the third month (EPB median = 3 days; NBF median = 9.5 days) and the sixth month (EPB median = 3 days; NBF median = 8.5 days) (Fig. 2).

**Fig. 3:**
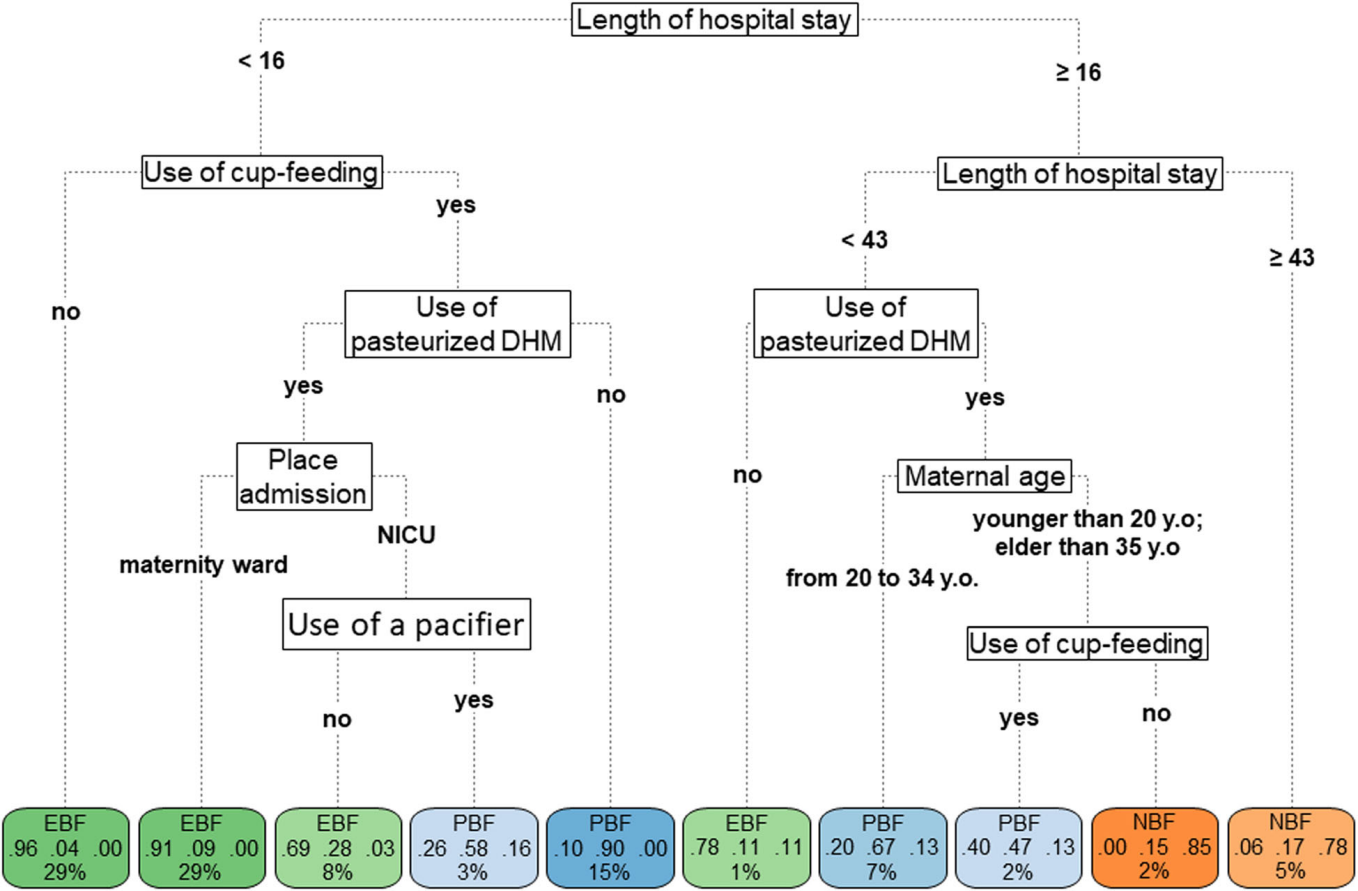
Decision-tree of 757 children at hospital discharge, Rio de Janeiro, Brazil, 2018. Note: EBF = Exclusive Breastfeeding; PBF = Partial Breastfeeding. NBF = Non-Breastfed. DHM = Donor human milk. Y.O. = years old. NICU = Neonatal intensive care unit. The length of hospital stay was measured in days

**Fig. 4:**
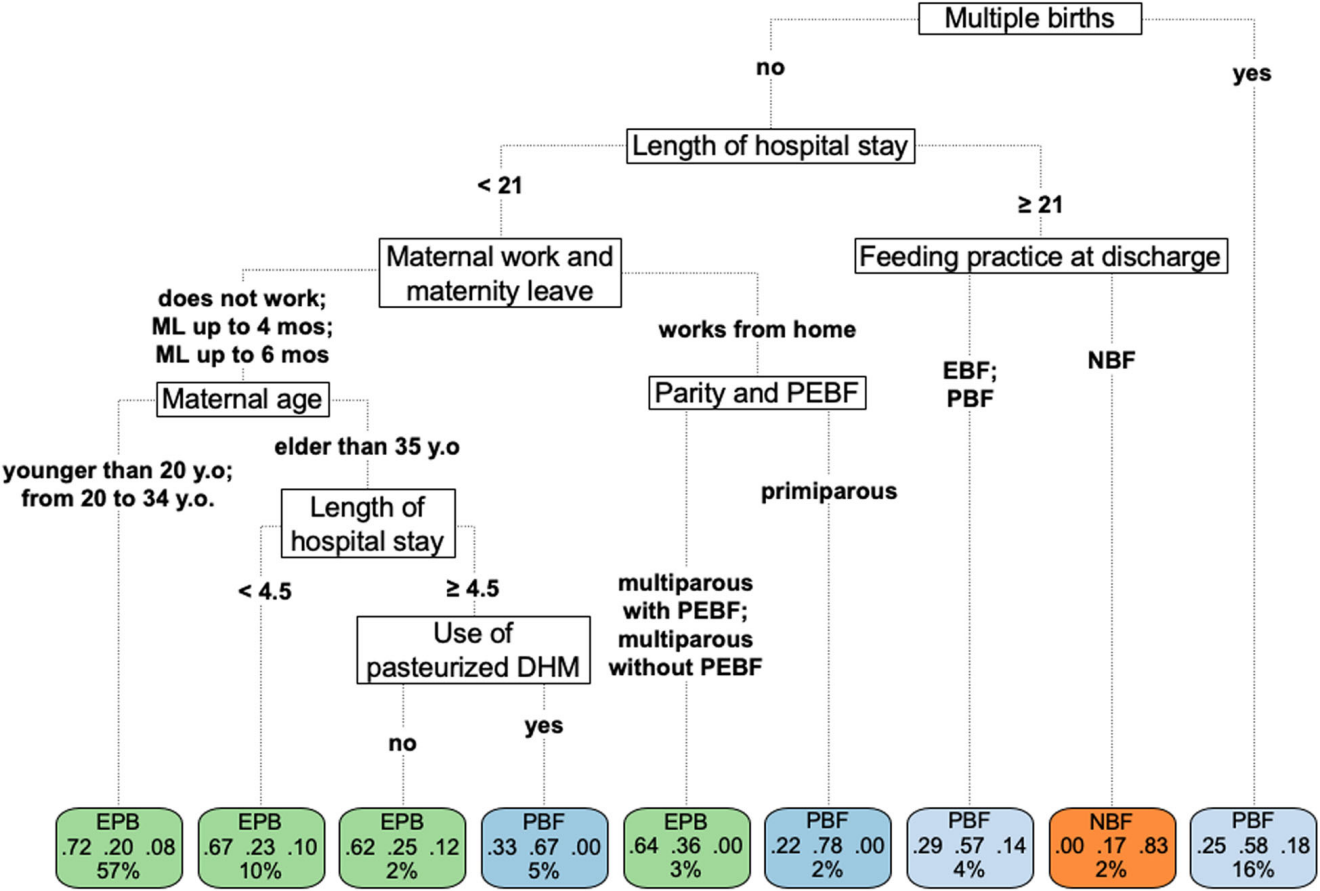
Decision-tree of 526 children at 3 months, Rio de Janeiro, Brazil, 2018. Note: EPB = Exclusive or Predominant Breastfeeding. PBF = Partial Breastfeeding. NBF = Non-Breastfed. DHN = Donor human milk. ML = Maternity leave. Y.O. = years old. PEBF = Previous experience of breastfeeding. The length of hospital stay was measured in days

**Fig. 5:**
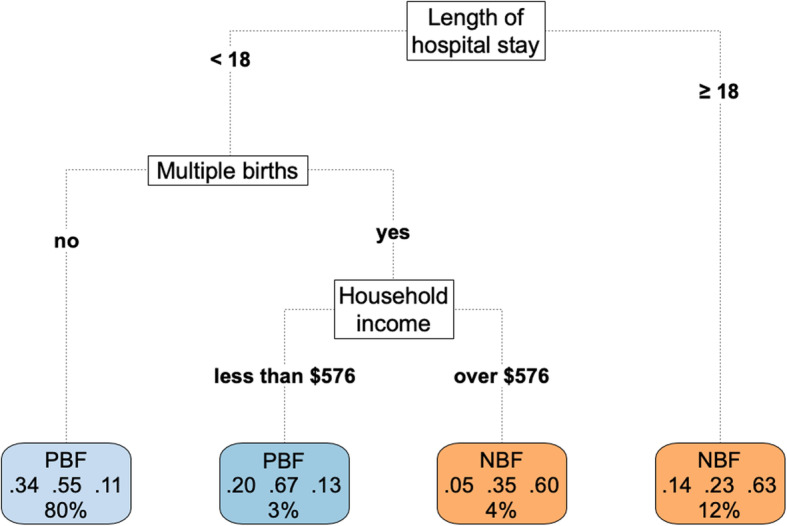
Decision tree of 459 children at 6 months, Rio de Janeiro, Brazil, 2018. Note: Household income (expressed in comparison to a reference value of two Brazilian monthly minimum wages at the time of the perinatal interview). ‘Minimum wage’ refers to the monthly minimum wage, as established by law, for formal employees in Brazil. [http://www.planalto.gov.br/ccivil_03/_Ato2015-2018/2016/Decreto/D8948.htm]; [http://receita.economia.gov.br/orientacao/tributaria/declaracoes-e-demonstrativos/ecf-escrituracao-contabil-fiscal/taxas-de-cambio-incluindo-valor-do-dolar-para-fins-fiscais-irpj-AC-anteriores]. PBF=Partial breastfeeding. NBF = Non-Breastfed. The length of hospital stay was measured in days

The mean accuracy of the fitted model on 10-fold cross-validation of the decision tree for the feeding practice was 83% at discharge (Fig. 3), 63% at 3 months (Fig. 4), and 50% at 6 months (Fig. 5).

At hospital discharge, the decision tree defined the “length of hospital stay” as the most important predictor of breastfeeding practice. When considering a length of hospital stay shorter than 16 days, the highest prevalence of EBF was observed (96%) in newborns who were not cup fed; among infants who were cup fed and in a maternity ward, the prevalence of EBF was 91%; among those cup fed with pasteurized donor human milk and in the NICU, the EBF percentage dropped by 40 percentage points (pp) with the use of a pacifier (i.e., the rate for no use of a pacifier was 69% and that for use of a pacifier was 26%); and for children who were cup fed and did not receive pasteurized human milk, PBF was prevalent, at a rate of 90% at hospital discharge (Fig. 3).

The prevalence of EBF was 78%, among infants who stayed in the hospital for 16–42 days and were not fed with pasteurized human milk. Within the group that was fed pasteurized human milk, PBF was prevalent in among mothers aged 20–34 years (67%). Among the younger and older mothers, when cup feeding was used, PBF was highly prevalent (47%), followed by EBF (40%); and when cup feeding was not used, the exclusive use of infant formula was prevalent (85%), where only 15% were still breastfed at hospital discharge.

Regarding the length of hospital stay of 43 days or more, NBF was prevalent at hospital discharge (78%), a branch not explained by any other predictor (Fig. 3).

In the third month of life, four variables that did not explain breastfeeding at hospital discharge were identified in the decision tree: “multiple births,” “maternal work and maternity leave,” “parity and previous experience of breastfeeding,” and “feeding practice at discharge.” The infants were divided into nine groups determined by eight nodes with 63% accuracy. EPB practice was predominant in four groups, comprising 72% of the participants. The probability of EPB ranged from 0 to 72% among the nine groups. The length of hospital stay remained an important predictor of the outcome, and multiples at births was highlighted as the most important predictor.

Among newborns who were multiples at births, PBF was frequent (58%), followed by EPB (25%). In singleton births with length of hospital stay shorter than 21 days, EPB was prevalent (varying from 22 to 72%) for any working condition, maternal age, parity, and when there was no supplementation with pasteurized donor human milk during the hospital stay. However, among women who worked at home, there was a drop in the prevalence of EPB among primiparous women as compared to among multiparous women (22 and 64%, respectively). The drop in EPB was also observed among infants born to older women (aged 35 years or older) who had been hospitalized for a period from 4 to 20 days and among infants supplemented with pasteurized human milk during the hospital stay. In this group of infants, the probability of EPB was half of that of the group that was not supplemented with pasteurized human milk (33 and 62%, respectively) (Fig. 4).

Hospital stay duration of 21 days or longer resulted in a low prevalence of EPB in the third month of life, varying from 0 to 29%. In this branch, breastfeeding was maintained in infants who were exclusively or partially breastfed at hospital discharge although most of them had already received infant formula (57%). The full discontinuation of breastfeeding, along with the use of infant formula, during the hospital stay resulted in the absence of EPB (0%) and a high prevalence of NBF (83%) (Fig. 4).

In the sixth month, the most accurate tree (54%) indicated that the length of hospital stay was the sole predictor of breastfeeding, and PBF and NBF were prevalent among children with a length of hospital stay of, respectively, < 18 days and ≥ 18 days. The second most accurate tree (50%) in the cross-validation analysis and with at least two predictive determinants is the one presented in Fig. 4. Infants were divided into four groups, formed by three nodes. Most of the sample belonged to two groups in which PBF was prevalent (83% of the participants). The probability of EPB ranged from 5 to 34% in the four groups.

In the sixth month of life, the length of hospital stay was still the most relevant predictor of feeding practice (the root node) as shown by the data. Among infants with a length of hospital stay shorter than 18 days, the prevalence of EPB varied from 5 to 34%; in the group of non-multiple pregnancies, PBF was prevalent (55%) followed by EPB (34%); in cases of multiple pregnancies, the change from PBF to NBF was found to be motivated by the increment in income and the prevalence of EPB dropped from 20 to 5%; and among infants with a long duration of hospital stay (of 18 days or longer), NBF was prevalent, and EPB was 14% (Fig. 5).

## Discussion

The prevalence of EBF was 65.2% at discharge; 51% at 3 months; and 20.6% at 6 six months. It is important to highlight 48.6% of the infants continued breastfeeding (PBF) in the sixth month. In the studied cohort, the analyzed components affected the risk prediction in different ways at different moments of an infant’s life (at hospital discharge, at 3 and at 6 months). In the three periods mentioned above, the length of hospital stay was relevant to the feeding practice. Besides the mother’s and child’s characteristics (multiples at births, maternal age, and parity), the social context, work, feeding practice during hospitalization, and several hospital practices and policies on breastfeeding influence the breastfeeding rates.

The length of hospital stay, a highlighted component in all periods, is a proxy for the severity of the child’s situation and the effectiveness of the provided care. The mother-infant separation [15, 16] may interfere with the recovery and negatively impact the hospital stay period [17]. Preterm newborns with low birthweight generally have long lengths of hospital stay that increase their vulnerability to negative outcomes and potentially affect the life trajectory of survivors [17, 18].

Previous studies [3, 19, 20] have shown that neonates with prolonged length of hospital stay are less likely to be breastfed than those with short lengths of stay. Thus, long lengths of hospital stay must involve a detailed exposition of hospital practices and special breastfeeding support and guidance to mothers of high-risk newborns in order to improve breastfeeding rates. Some studies show that the greater the rate of breastfeeding in the NICU, the shorter the length of hospital stay [21] and the higher the cost savings [22, 23].

This study highlights the need to implement hospital practices to promote breastfeeding in hospitals that care for high-risk newborns and support the expansion of the BFHI and efforts within the scope of public health policies to ensure that human milk banks (HMBs) fulfill their role as agents of promotion, protection, and support for breastfeeding (with special emphasis on the risk segment of neonatal care), so that a long hospital does not adversely affect the rates of breastfeeding.

On evaluating the hospital stay tree, the change in the predominance of breastfeeding practice from EBF to PBF only regarded the use of a pacifier among neonates hospitalized in the NICU, and the change from PBF to NBF regarded the non-use of cup feeding among infants with long lengths of hospital stay. The use of a pacifier and the non-use of cup feeding of human milk were predictors that negatively affected breastfeeding in the group of newborns who received supplementation with pasteurized donor human milk.

During hospital stay, some components may facilitate or hinder the early establishment of EBF. Our results are similar to those of other findings regarding the use of cup feeding, which improves EBF rates at discharge, even in preterm babies and those with low birthweight [24–26]. This may be due to the similarity in the muscle activity in the orofacial region of infants who are breastfed and cup fed [27, 28].

Our data show that the use of human milk during the length of hospital stay resulted in EBF at discharge. When supplements are required or desired, human milk provided by the mother [29] or by an HMB [30] offers several benefits to hospitalized high-risk newborns [2, 21, 31–33]. There are well-documented general and systemic benefits [1] as well as specific benefits of human milk for high-risk newborns, such as protection from necrotizing enterocolitis, retinopathy of prematurity, and bronchopulmonary dysplasia, among others [33–35]. All these specific benefits also impact the length of hospital stay.

The use of a pacifier was found to be a predictor of early termination of EBF at discharge. Studies have shown that the use of a pacifier may be a risk factor for the early discontinuation of EBF [4, 36, 37] and that the association is related to the time it was introduced and the frequency of use [38]. This happens even among mothers who are highly motivated to breastfeed [39]. Minimizing the use of a pacifier during the transition process of the newborn from tube feeding to breastfeeding is associated with early exclusive breastfeeding [3, 40].

Breastfeeding practice during hospital stay was one of the major predictors of the continuation of this behavior in the third month. A recent study [1, 41] adapted the determinants of breastfeeding practice by highlighting the chronology of breastfeeding indicators; the study showed that to ensure consistent practice, the practice must be followed at different moments (from the establishment of this practice in the first hour to the second year of life).

Another important predictor in the third and sixth months was multiple pregnancy. A previous study [42] showed that twin newborns are not breastfed at the same rate as single newborns and have a higher risk of early weaning.

A change in the feeding practice was noticed in the decision tree in the third month in relation to hospital discharge and supplementation with human milk (during hospital stay). In order to better understand this prediction, the characteristics of 24 children in this group were explored (average length of hospital stay = 9 days): 15 were born with perinatal morbidity, 4 were preterm, 13 remained hospitalized in the maternity ward, 11 remained hospitalized in the NICU, none of them used a pacifier during hospital stay, 22 were cup fed during hospital stay, 16 did not have skin-to-skin contact, and 13 mothers had difficulties in breastfeeding in the last month.

Feeding supplementation negatively interferes with the decision to breastfeed, especially in primiparous or elder women (35 years old or over) [43]. Once supplements are introduced during the length of hospital stay, regardless of the type of milk prescribed, women start questioning their capacity to breastfeed [44]. As a result, there is a high tendency to offer supplements at home. The advice and practices of healthcare professionals influence breastfeeding practices [1].

Long length of hospital stay was a predictor of EBF discontinuation [3, 19, 20]. When there is risk or potential risk at birth, the longer length of hospital stay must be used to expose the mother-infant dyad to favorable hospital practices for breastfeeding [3]. Besides the generic know-how of the healthcare providers, high-level expertise in breastfeeding, experience, and specific skills are the foundations of proper management of vulnerable neonates.

The exclusive breastfeeding rates under 6 months in a high-risk setting were not correlated with overall national breastfeeding rates. The prevalence of EBF in Brazil was approximately 40% among infants aged under 6 months [45]. In this study, the prevalence of EBF at 6 months was 20.6%, which is slightly higher than the prevalence of 14.5% observed in the Pelotas cohort [46] and of 13% in the cohort of preterm babies in Denmark [47]. In the present study, the prevalence of EBF among high-risk newborns was similar to that among low-risk newborns reported in previous studies. Breastfeeding competence and behavior are not developed by factors such as the presence or absence of risk at the time of birth, but instead, they are affected by several determinants related to the mothers, infants, health systems and services, and healthcare providers. The breastfeeding rates in the highlighted studies, although similar to each other, are below international recommendations [48].

Income was found to be a predictor of the analyzed outcomes only in the sixth month. Partial breastfeeding was more common among poorest mothers with multiple pregnancies than among mothers with a household income higher than twice the monthly minimum wages (over $576). Financially well-equipped mothers are highly likely to use formulas as a result of marketing pressure and economic well-being [46].

We built an analysis model that provided a robust classification of factors predicting the feeding practice for each infant with an accuracy ranging between 50 and 83%, so it can be used for quick decision making. Although prediction models for breastfeeding have been developed and widely applied, most of them are based almost exclusively on parametric or semi-parametric statistical methods, which rely on restrictive model assumptions. In this paper, we proposed the use of a decision-tree method, which is a completely nonparametric machine learning method for accurate prediction. In addition, in clinical practice, decision trees may be a suitable alternative to traditional statistical methods, since they allow the analysis of interactions between various risk components, including those not known previously. Therefore, this study ranked a set of predictors for the statistical modeling of breastfeeding determinants in hospitals that care for high-risk newborns. The predictive capacity of the model described was linked to the pre-processing techniques carefully adopted in the data analysis stage and sought to deal with problems such as missing data, outliers, and multicollinearity of predictor variables.

As far as we know, this longitudinal study is among the few based on data about breastfeeding rates in high-risk hospitals in Latin America. This is the first Brazilian study that applied machine learning models to predict breastfeeding in a cohort of infants delivered at a high-risk hospital.

The main limitation of this analysis was the selection bias related to the social determinants. The support network was not assessed in this study, which could possibly explain some results. Another limitation refers to the joint analysis of the categories “predominant breastfeeding” and “exclusive breastfeeding” due to the low frequency in the former (7 and 9% in the third and sixth months, respectively). Another limitation could be that public health hospitals mainly serve the low-income population, despite free, universal healthcare being available for all citizens since the creation of the Unified Health System (SUS) in 1988 by the Brazilian Federal Constitution. However, this pattern was not confirmed in our study, since more than half of the participants (60%) had a household income higher than $576 a month, most likely because of the fact that this hospital is a national referral center for high-risk infants. It is relevant to mention that these outcomes pertain to a single center and may not be suitable for generalization to the larger population in Brazil or in other countries.

## Conclusions

This study provides a better understanding of the predictors of breastfeeding cessation in settings with a wide range of expositions. This study found that the length of hospital stay was the main determinant of breastfeeding practice throughout the 6 months of life, and multiple pregnancy was an important predictor of this practice in the third and sixth months. Individual determinants, based on social context, employment prospects, breastfeeding practice during hospitalization, and the health system were important predictors of this practice.

The combination algorithm of the decision trees is a practical tool that can be used to predict the groups at risk of early discontinuation of EBF and provide effective and timely interventions in order to ensure prolonged and high rates of breastfeeding.

Our results suggest that implementing breastfeeding promotion policies in hospitals for high-risk infants can help overcome the difficulties related to breastfeeding among these infants. Our findings may also provide a basis for country-level recommendations for this population.

## Supplementary Information

**Additional file 1.** Comparison between included and excluded participants due to missing data. Rio de Janeiro, Brazil, 2018.

## Data Availability

All relevant data are in this paper. The datasets generated and/or analyzed during the current study are not publicly available due to guidelines from The Ethics Committees, to restrict the full data disclosure since this could compromise participant confidentiality. However, research data are available from the corresponding author on reasonable request. We welcome data analysis and publication collaboration through specific research proposals sent to the lead researcher and her co-tutors. Additional information can be obtained by sending an e-mail to maira.silva@iff.fiocruz.br

## Abbreviations

EBF: Exclusive Breastfeeding
EPB: Exclusive or Predominant Breastfeeding
PBF: Partial Breastfeeding
FIOCRUZ: Oswaldo Cruz Foundation
HIV: Human Immunodeficiency Virus
HMB: Human Milk Bank
HTLV: Human T-cell Lymphotropic Virus
IFF: National Institute of Women, Children and Adolescents Health Fernandes Figueira
NBF: Non-Breastfed
NICU: Neonatal Intensive Care Unit
WHO: World Health Organization
