# Supplementary material for: Predicting risk of early discontinuation of exclusive breastfeeding at a Brazilian referral hospital for high-risk neonates and infants: a decision-tree analysis
Source: Int Breastfeed J. 2021 Jan 4;16:2. doi: 10.1186/s13006-020-00349-x (PMC7783998; doi:10.1186/s13006-020-00349-x)
Supplement: Supplementary file 1 — Additional file 1. Comparison between included and excluded participants due to missing data. Rio de Janeiro, Brazil, 2018. [file 13006_2020_349_MOESM1_ESM.docx]

**Additional file 1. Comparison between included and excluded participants due to missing data. Rio de Janeiro, Brazil, 2018.**

| **Characteristics** |  | **Hospital discharge n (%)** | |  | **3 months of age n (%)** | |  | **6 months of age n (%)** | |
| --- | --- | --- | --- | --- | --- | --- | --- | --- | --- |
|  |  | **included (N=757)** | **excluded (N=246)** |  | **included (N=526)** | **excluded (N=191)** |  | **included (N=459)** | **excluded (N=171)** |
| Multiple births | no | 635 (83.9) | 219 (89.0) |  | 441 (83.8) | 153 (80.1) |  | 410 (89.3) | 153 (89.5) |
|  | yes | 122 (16.1) | 27 (11.0) |  | 85 (16.2) | 38 (19.9) |  | 49 (10.7) | 18 (10.5) |
| Gestational age | >37 | 590 (77.9) | 187 (76.0) |  | 424 (80.6) | 151 (79.1) |  | 381 (83.0) | 140 (81.9) |
|  | < 37 | 167 (22.1) | 59 (24.0) |  | 102 (19.4) | 40 (20.9) |  | 78 (17.0) | 31 (18.1) |
| Birth weight | <1500g | 21 (2.8) | 17 (6.9) |  | 10 (1.9) | 10 (5.2) |  | 11 (2.4) | 6 (3.5) |
|  | 1500-2500g | 120 (15.9) | 39 (15.9) |  | 80 (15.2) | 23 (12.0) |  | 61 (13.3) | 16 (9.4) |
|  | >2500g | 616 (81.4) | 190 (77.2) |  | 436 (82.9) | 158 (82.7) |  | 387 (84.3) | 149 (87.1) |
| Perinatal morbidity | no | 443 (58.5) | 143 (58.1) |  | 329 (62.5) | 123 (64.4) |  | 290 (63.2) | 114 (66.7) |
|  | yes | 314 (41.5) | 103 (41.9) |  | 197 (37.5) | 68 (35.6) |  | 169 (36.8) | 57 (33.3) |
| Maternal age^a,c^ | younger than 20 years old | **78 (10.3)** | **61 (24.8)** |  | **47 (8.9)** | **35 (18.5)** |  | **43 (9.4)** | **38 (22.5)** |
|  | from 20 to 34 years old | **533 (70.7)** | **154 (62.6)** |  | **373 (70.9)** | **124 (65.6)** |  | **318 (69.3)** | **105 (62.1)** |
|  | elder than 35 years old | **143 (19.0)** | **31 (12.6)** |  | **106 (20.2)** | **30 (15.9)** |  | **98 (21.4)** | **26 (15.4)** |
| Maternal education^a,b,c^ | up to elementary school | **249 (33.0)** | **134 (54.5)** |  | **157 (29.8)** | **89 (47.1)** |  | **125 (27.2)** | **83 (49.1)** |
|  | secondary school or higher | **505 (67.0)** | **112 (45.5)** |  | **369 (70.2)** | **100 (52.9)** |  | **334 (72.8)** | **86 (50.9)** |
| Household income^d^ | less than $576 | 294 (39.0) | 31 (44.9) |  | 199 (37.8) | 35 (41.7) |  | 171 (37.3) | 31 (40.3) |
|  | over $576 | 460 (61.0) | 38 (55.1) |  | 327 (62.2) | 49 (58.3) |  | 288 (62.7) | 46 (59.7) |
| Parity and PEBF | primiparous | 373 (49.5) | 120 (53.8) |  | 259 (49.2) | 96 (55.8) |  | 231 (50.3) | 86 (56.2) |
|  | multiparous with PEBF | 342 (45.4) | 94 (42.2) |  | 237 (45.1) | 67 (39.0) |  | 201 (43.8) | 56 (36.6) |
|  | multiparous without PEBF | 39 (5.2) | 9 (4.0) |  | 30 (5.7) | 9 (5.2) |  | 27 (5.9) | 11 (7.2) |
| Maternal work and maternity leave^a,b,c^ | does not work | **375 (49.7)** | **170 (73.6)** |  | **262 (49.8)** | **118 (65.2)** |  | **226 (49.2)** | **109 (68.6)** |
|  | works from home | **37 (4.9)** | **5 (2.2)** |  | **27 (5.1)** | **4 (2.2)** |  | **23 (5.0)** | **3 (1.9)** |
|  | works with 6-month ML benefits | **39 (5.2)** | **1 (0.4)** |  | **32 (6.1)** | **4 (2.2)** |  | **27 (5.9)** | **2 (1.3)** |
|  | works with 4-month ML benefits | **210 (27.9)** | **35 (15.2)** |  | **141 (26.8)** | **40 (22.1)** |  | **124 (27.0)** | **33 (20.8)** |
|  | works without ML benefits | **93 (12.3)** | **20 (8.7)** |  | **64 (12.2)** | **15 (8.3)** |  | **59 (12.9)** | **12 (7.5)** |
| Tobacco use during pregnancy | no | 694 (92.0) | 220 (90.9) |  | 494 (93.9) | 172 (93.0) |  | 436 (95.0) | 153 (91.6) |
|  | yes | 60 (8.0) | 22 (9.1) |  | 32 (6.1) | 13 (7.0) |  | 23 (5.0) | 14 (8.4) |
| Place of hospital admission | maternity ward | 526 (69.5) | 159 (64.9) |  | 384 (73.0) | 139 (73.2) |  | 333 (72.5) | 122 (71.8) |
|  | neonatal intensive care unit | 231 (30.5) | 86 (35.1) |  | 142 (27.0) | 51 (26.8) |  | 126 (27.5) | 48 (28.2) |
| Feeding practice at hospital discharge | EBF | 495 (65.4) | 146 (64.6) |  | 358 (68.1) | 126 (67.0) |  | 308 (67.1) | 109 (64.9) |
|  | PBF | 208 (27.5) | 62 (27.4) |  | 145 (27.6) | 51 (27.1) |  | 124 (27.0) | 51 (30.4) |
|  | NBF | 54 (7.1) | 18 (8.0) |  | 23 (4.4) | 11 (5.9) |  | 27 (5.9) | 8 (4.8) |

Note: a= Difference> 10 percentage points at hospital discharge between included and excluded participants; b= Difference> 10 percentage points at 3 months between included and excluded participants; c= Difference> 10 percentage points at 6 months between included and excluded participants; d= Household income (expressed in comparison to a reference value of two Brazilian monthly minimum wages at the time of the perinatal interview). ‘Minimum wage’ refers to the monthly minimum wage, as established by law, for formal employees in Brazil. [http://www.planalto.gov.br/ccivil_03/_Ato2015-2018/2016/Decreto/D8948.htm]; [<http://receita.economia.gov.br/orientacao/tributaria/declaracoes-e-demonstrativos/ecf-escrituracao-contabil-fiscal/taxas-de-cambio-incluindo-valor-do-dolar-para-fins-fiscais-irpj-AC-anteriores>].

Bold percentages refer to statistical significance (p-value <0.005) and were based on Chi-square and Fisher tests.

PEBF=previous experience of breastfeeding. ML= maternity leave. EBF=exclusive breastfeeding. PBF=partial breastfeeding. NBF=non-breastfed.
